# A Systematic Review of Tuberculosis Stigma Reduction Interventions

**DOI:** 10.3390/healthcare13151846

**Published:** 2025-07-29

**Authors:** Nadira Aitambayeva, Altyn Aringazina, Laila Nazarova, Kamila Faizullina, Magripa Bapayeva, Nazerke Narymbayeva, Shnara Svetlanova

**Affiliations:** 1 Department of Public Health and Social Sciences, Kazakhstan’s Medical University “KSPH”, Almaty 050000, Kazakhstan; 2 School of Health Sciences, Almaty Management University AlmaU, Almaty 050060, Kazakhstan; altyn.aringazina@gmail.com; 3 Department of Epidemiology, Evidence-Based Medicine and Biostatistics, Kazakhstan’s Medical University “KSPH”, Almaty 050000, Kazakhstan; zauirbekovna92@gmail.com; 4 Almaty City Branch of the Salidat Kairbekova National Research Center for Health Development, Almaty 050010, Kazakhstan; faizullina.kamila80@gmail.com; 5 Department of Internal Medicine, Kazakhstan’s Medical University “KSPH”, Almaty 050000, Kazakhstan; magripa.bapaeva@gmail.com; 6 Department of Health Management, Kazakhstan Medical University “KSPH”, Almaty 050000, Kazakhstan; n.narymbay@gmail.com; 7 Department of Nursing, Kazakhstan’s Medical University “KSPH”, Almaty 050000, Kazakhstan; svetlanova.sh@kaznmu.kz

**Keywords:** TB, evaluation, discrimination, cascade of care

## Abstract

Background: Stigma associated with tuberculosis (TB) continues to undermine patient well-being, treatment adherence, and public health goals and objectives. This study aims to systematically review the literature to identify and synthesize TB stigma reduction interventions published between 2015 and 2025. Methods: Following the PRISMA guidelines, we conducted a comprehensive literature search across PubMed, Scopus, Science Direct, ProQuest, and Google Scholar. Eligible studies included those with qualitative, quantitative, and mixed-methods designs that focused on interventions related to TB-related stigma. We categorized the studies into three groups: (1) intervention development studies, (2) TB treatment programs with stigma reduction outcomes, (3) stigma-specific interventions. Data extraction and quality appraisal were conducted independently by two reviewers using the Mixed Methods Appraisal Tool (MMAT). Results: A total of 15 studies met the inclusion criteria. Five studies focused on co-developing stigma interventions, which incorporated multi-level and multicomponent strategies targeting internalized, enacted, anticipated, and intersectional stigma. Two studies assessed TB treatment-related interventions (e.g., home-based care, digital adherence tools) with incidental stigma reduction effects. The remaining seven studies implemented stigma-targeted interventions, including educational programs, video-based therapy, peer-led support, and anti-self-stigma toolkits. Interventions addressed stigma across individual, interpersonal, institutional, community, and policy levels. Conclusions: This review highlights the evolution and diversification of TB stigma interventions over the past decade. While earlier interventions emphasized education and support, recent strategies increasingly integrate peer leadership, digital platforms, and socio-ecological frameworks. The findings underscore the need for comprehensive, contextually grounded interventions that reflect the lived experiences of people affected by TB.

## 1. Introduction

Tuberculosis (TB) remains a significant global health burden, with annual incidence exceeding 10 million cases [1]. The Global TB Report (2024) [1] suggests that TB regained its position as the leading cause of infectious disease mortality in 2023, after being temporarily surpassed by COVID-19 during the preceding three-year period. TB incidence exhibits significant socioeconomic disparities, with the highest disease burden concentrated in low-income populations and marginalized communities [2]. Social and structural determinants of health significantly influence TB-related stigma [3], which remains prevalent, affecting approximately 34% of individuals with infectious diseases, with higher rates observed in low- and middle-income countries [4]. Despite the availability of effective diagnostics and treatment, TB control continues to be hampered by pervasive stigma, which compromises health-seeking behavior, treatment adherence, and psychosocial well-being [5]. Stigma not only impedes timely diagnosis and disclosure of TB status but also leads to social isolation, discrimination, and economic constraints [6,7]. Emerging evidence indicates that healthcare provider stigma may influence treatment modality selection [8]. These consequences are particularly pronounced among individuals with drug-resistant TB (DR-TB), those with a history of incarceration, and people residing in settings with entrenched structural inequalities [9].

TB-related stigma is complex and multidimensional, encompassing internalized, anticipated, enacted, perceived, and secondary stigma [10]. It is driven by a combination of cultural beliefs, misinformation, fear of contagion, and health system practices that inadvertently reinforce negative stereotypes [11]. Importantly, stigma operates across multiple socio-ecological levels—from the individual and interpersonal to institutional, community, and policy domains—necessitating interventions that are not only multicomponent but also contextually relevant and equity-focused. Hence, stigma reduction represents an essential component of successful TB eradication efforts [12].

Growing recognition of TB stigma as a barrier to achieving global TB elimination goals has prompted increased attention to stigma reduction in national and international TB strategies [13]. The Global Plan to End TB 2023–2030, developed by the Stop TB Partnership, identifies stigma and discrimination reduction as a key implementation component [14]. TB stigma is well studied, and several reviews have examined available interventions. A review on drug-resistant TB highlighted three priorities for protecting healthcare workers: strengthening infection control, improving staff competence, and providing psychosocial support [15]. A systematic review of TB stigma interventions in developing economies found that optimal implementation depends on the healthcare setting, available resources, and local TB epidemiology [16]. In 2017, Sommerland and colleagues published a systematic review of TB stigma interventions, highlighting the limited number of rigorously evaluated interventions and a lack of theoretical grounding in many existing programs [17]. Since then, a broader range of interventions has emerged. These developments call for a systematic reassessment of the current landscape of TB stigma interventions to inform evidence-based programming and future research directions.

This study addresses the existing knowledge gap by providing an updated synthesis of the evidence base from 2015 to 2025. This systematic review aims to analyze TB stigma intervention components across: (a) TB stigma intervention co-development programs, (b) TB treatment interventions with stigma effects, (c) dedicated stigma reduction strategies in TB care.

## 2. Materials and Methods

### 2.1. Search Strategy, Study Selection, and Data Collection

We conducted a systematic review following the Preferred Reporting Items for Systematic Reviews and Meta-Analyses (PRISMA) guidelines [14], updating the evidence on TB stigma reduction interventions since the 2017 systematic review on this topic. The PRISMA 2020 checklist is available as Supplementary Table S1.

The review protocol was registered with the PROSPERO International prospective register of systematic reviews (ID: CRD420251058317) after confirming no overlapping reviews existed. We searched PubMed, Scopus, Science Direct, ProQuest, and Google Scholar for English-language studies published between January 2015 and April 2025. The 2015 cutoff was selected because earlier evidence had been included in the 2017 review. The final search strategy combined the following keywords: “tuberculosis” OR “TB” AND “stigma” AND (“intervention” OR “reduction”). The detailed search strategy is presented in Table 1.

Table 2 presents the eligibility criteria following the Population, Intervention, Comparator, Outcome, and Study Design (PICOS) framework, with the population encompassing the general public, adolescents, TB patients/survivors, caregivers, and healthcare providers while excluding HIV patients and HIV/TB co-infected individuals. Eligible interventions included stigma-focused intervention/program development, TB treatment interventions demonstrating stigma reduction, and dedicated stigma reduction initiatives, excluding TB treatments without assessed stigma impacts. No comparator criteria were applied. Outcomes focused on stigma types addressed, target populations, and intervention components, excluding studies reporting only TB treatment efficacy or knowledge outcomes without stigma metrics. The review incorporated qualitative, quantitative, and mixed-methods studies but excluded reviews, editorials, commentaries, conference abstracts, and non-English publications.

Eligibility assessment and data collection were conducted following PRISMA guidelines [14]. Two independent researchers (N.A. & A.A.) performed a two-stage screening process: initial title/abstract screening followed by full-text evaluation. After implementing the search strategy, the identified records were consolidated in Mendeley reference manager, with duplicate records removed. Unique records underwent title/abstract screening for relevance, followed by full-text assessment against predefined inclusion/exclusion criteria.

Using a standardized data extraction form, we collected the following data: first author’s name, publication year, country of origin, study design, methodology, target population, outcomes, sample size, stigma type(s) assessed, stigma scale, stigma assessment results, and intervention details (where applicable). Following independent extraction, the two researchers (N.A. & A.A.) compared datasets and resolved discrepancies through iterative discussion with a third author (S.S.) until consensus was achieved for all included studies and extracted data. We provide information on the stigma assessment tools, along with mean and standard deviation values for the stigma scales from the included studies, in Supplemental Table S2.

### 2.2. Data Synthesis

For data synthesis, to meet the aim of our study, we categorized the stigma interventions into three groups: (1) TB stigma intervention development studies—projects that design, pilot, or validate effective approaches to reduce TB stigma. (2) TB treatment interventions with stigma outcomes—clinical or adherence programs whose primary aim is treatment success, but that also measure and report quantitative reductions in stigma. (3) Targeted stigma reduction interventions—programs whose main objective is to lessen TB-related stigma, whether among TB patients or the wider community. Findings are organized in tables by stigma type addressed, intervention approach, and relevant components.

### 2.3. Risk of Bias Evaluation

The Mixed Methods Appraisal Tool (MMAT) was used to evaluate the methodological quality of five study designs: qualitative research, quantitative randomized controlled trials (RCTs), quantitative non-randomized studies, quantitative descriptive studies, and mixed-methods studies. The assessment form consisted of seven questions rated as “yes,” “no,” or “can’t tell.” The first two questions applied to all study types and assessed whether the research questions were clearly stated and appropriately addressed. Study-type-specific questions with risk of bias assessment results are presented in Table 3. All included studies met at least five “yes” criteria on the MMAT, demonstrating satisfactory quality and low risk of bias, and were therefore retained for further analysis.

## 3. Results

**Figure 1:**
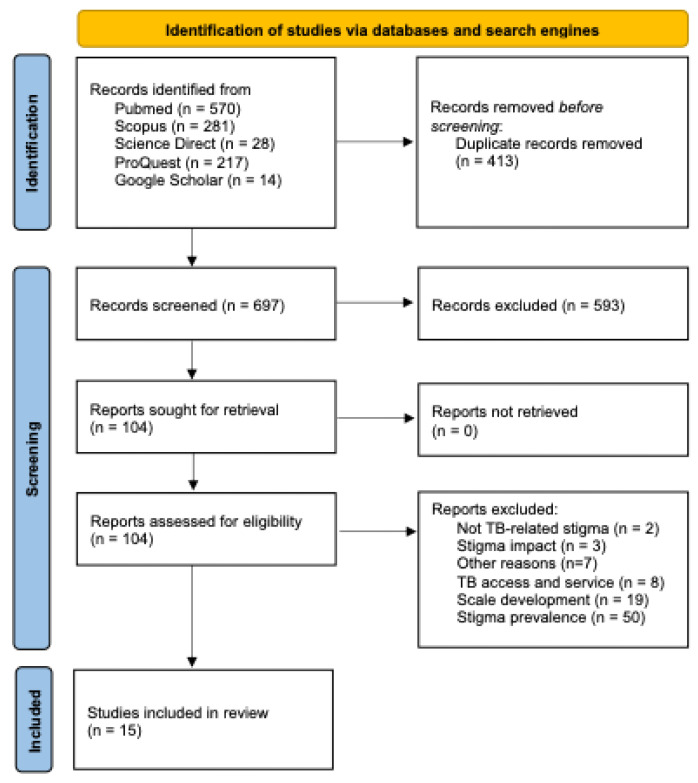
PRISMA flowchart of study identification and selection.

Our systematic search identified 1110 articles from databases and search engines based on the predefined search strategy. After removing duplicates, we screened 697 titles and abstracts, and selected 104 for full-text review. Following the full-text assessment, 15 studies met the PICOS eligibility criteria and were included in the review. The exclusions included two studies not focused on TB-related stigma, three studies that examined stigma impacts, seven studies excluded for miscellaneous reasons, eight focused solely on TB access/services, nineteen that addressed TB stigma scale development, and fifty that reported TB-stigma prevalence. Figure 1 [14] presents the PRISMA flowchart detailing the selection process.

Among the fifteen included studies, five focused on the development of stigma interventions, three studies presented results of tuberculosis treatment-related interventions with demonstrated impact on stigma reduction, while seven studies focused on stigma reduction interventions. The included studies were conducted across multiple countries, with the majority originating from South Asia and Sub-Saharan Africa—notably India (2 studies), Indonesia (3), Thailand (2), and South Africa (3), as well as Malaysia, Turkey, Somalia, El Salvador, and Uganda—reflecting a diverse geographical distribution of tuberculosis stigma reduction interventions. The studies employed a variety of methodological approaches, including qualitative methods, such as in-depth interviews, community-based participatory research, and case reports; mixed-methods designs, including participatory action research; and quantitative designs, such as randomized controlled trials (RCTs), quasi-experimental studies, human-centered design, cross-sectional analyses, longitudinal assessments, and one-group repeated-measure designs. Seven studies included only TB patients, and two studies included high school students, while the rest of the studies also included other community members and healthcare workers. Further details on the selected studies can be found in Table 4.

The systematic review identified five studies focused on the co-development of TB stigma interventions, each outlining a range of multi-level components tailored to address different types of stigma. Across these studies, interventions were consistently grounded in socio-ecological or health stigma frameworks and addressed internalized, anticipated, enacted, perceived, and intersectional stigma. Sabin (2021) [19] highlighted the use of celebrity advocacy and school-based education to address enacted stigma, while support groups and counseling were recommended to mitigate internalized stigma. Foster (2024) [24] emphasized community engagement and survivor-led messaging in multicomponent interventions. Hayward (2024) [22] offered a comprehensive structure, organizing stigma interventions across five socio-ecological levels—from individual (peer-led digital platforms and structured counseling) to institutional (training informed by survivor input) and policy levels (reform of stigmatizing health system practices). Similarly, van der Westhuizen (2024) [20] applied Link and Phelan’s framework to identify stigma mechanisms, proposing patient education, respectful communication of infection control measures, reframing of institutional policies, and implementation of universal airborne precautions as critical strategies. Fuady (2025) [25] proposed four peer- and community-based modules: psychological screening, peer-led group and individual counseling, and community-level TB awareness sessions.

When comparing these findings with studies where TB treatment-related interventions had a secondary stigma-reducing effect, fewer stigma-specific components were described. In their 2016 study, Wilson (2016) [23] examined how a video-based TB education program influenced (a) the knowledge/attitudes of patients and families, (b) manifestations of TB-associated stigma. Taneja (2017) [26] implemented a home-based care model incorporating counseling, rehabilitation, and nutritional support alongside pharmacological treatment, reporting reductions in enacted stigma. Patel (2020) [18] described a human-centered redesign of the 99DOTS digital adherence platform that focused on improving user experience and minimizing stigma through privacy-sensitive medication packaging.

In contrast, the third group of studies explicitly targeted stigma reduction. These interventions commonly combined education, peer support, and digital tools. For instance, Moonsarn (2023) [28] implemented a four-part education program targeting stigma knowledge, cognitive restructuring, and empowerment. Macdonald (2024) [29] developed an eight-module anti-self-stigma toolkit based on a socio-ecological model tailored for drug-resistant TB. Interventions such as that by Vibulchai (2024) [30] integrated health education with psychoeducation and self-management, delivered through peer facilitators and home visits. School-based educational programs as in Idris (2020), psychosocial workplace interventions as in Mawey (2023) [21], and video-based therapy as in Kara (2022) [27] and Faraade (2023) were also shown to reduce perceived, internalized, and enacted stigma through structured health communication and community engagement. Table 5 presents detailed information on the stigma types, intervention types, and components.

## 4. Discussion

This systematic review synthesized evidence from 15 studies published between 2015 and 2025 that addressed TB-related stigma through the co-development of novel interventions, the integration of stigma reduction into treatment programs, or the direct implementation of stigma reduction strategies. The findings revealed that TB stigma is a complex phenomenon, and that recent interventions have increasingly adopted multi-level, context-specific strategies. Notably, co-development studies frequently emphasized participatory design, peer involvement, and socio-ecological frameworks. Interventions that were not initially designed to address stigma, such as video-based education, home-based care, or human-centered technological redesigns, nevertheless demonstrated secondary benefits in stigma mitigation. Explicitly stigma-reduction-focused interventions included structured educational curricula, psychosocial counseling, digital adherence technologies, and peer-led outreach efforts, collectively representing the breadth of modalities available to address TB stigma.

This review demonstrates that the choice of target population strongly shapes both the design and the implementation pathways of TB stigma interventions. Programs targeting PWTB and their families typically incorporate individual-level psychosocial support and peer leadership, as these approaches directly address internalized and anticipated stigma. In contrast, interventions delivered to TB-unaffected audiences prioritize knowledge-building and norm-shifting strategies to curb enacted or community-level stigma. Studies centered on healthcare providers address organizational culture and infection-control communication to dismantle institutional stigma. Recognizing these population-specific pathways can help researchers in aligning intervention components with the dominant stigma mechanisms in each context.

The present findings build upon and expand the scope of the systematic review by Sommerland et al. (2017) [17], which identified only seven eligible studies prior to 2015. While earlier research predominantly targeted stigma at the individual level—emphasizing patient knowledge and awareness—the current body of evidence reflects a paradigm shift toward addressing the structural and systemic roots of stigma. Notably, recent studies by Hayward (2024) [22], Foster (2024) [24], and van der Westhuizen (2024) [20] employed intersectional stigma frameworks and socio-ecological models to design interventions spanning multiple domains, including the individual, interpersonal, institutional, community, and policy levels. These multi-level strategies represent a maturation of the field, moving beyond unidimensional approaches to embed stigma reduction within broader health systems and sociopolitical structures. Such integration is essential for addressing not only internalized stigma, but also the more entrenched forms of enacted and structural stigma that limit access to diagnosis, care, and social support [10]. Research suggests that TB stigma emerges through sociostructural processes, wherein macroeconomic factors, health policies, and service delivery mechanisms interact to generate experiences of both anticipated and internalized stigma among affected populations [33].

Effective initiatives aimed at reducing stigma emphasize the crucial role of community involvement, awareness campaigns, and professional development for healthcare providers in creating a more supportive and inclusive space for those affected [34,35]. Previous analyses of intervention pathways for TB stigma reduction have proposed a framework targeting four key groups simultaneously: the general population, healthcare providers, TB patients, and TB survivors [36]. This review expands the evidence base by identifying stigma reduction interventions that either target TB patients and the public concurrently, or address both healthcare providers and TB patients simultaneously. Moreover, by delineating the specific active components through which these interventions achieve stigma reduction, this review offers a strategic roadmap for designing and evaluating future interventions. Empirical research and programmatic experience converge to demonstrate that sustainable TB elimination is contingent upon effective stigma reduction [13].

Several successful policy- and community-based initiatives illustrate the feasibility and impact of stigma reduction in TB care. For example, India’s National Strategic Plan for TB Elimination incorporated a stigma reduction training curriculum co-developed by KHPT and USAID, which was piloted in Karnataka and Telangana and later scaled nationally through the “Breaking the Barriers” project [37]. This initiative trained healthcare workers and community leaders to recognize and address stigma, leading to improved patient–provider interactions and increased treatment uptake. Additionally, a scoping review by Anindhita et al. (2024) identified community-based psychosocial support interventions—such as peer-led counseling and support groups—as effective in reducing internalized and anticipated stigma among TB-affected populations [38]. These examples underscore the importance of embedding stigma reduction into national policy frameworks and leveraging community assets to foster inclusive TB care environments.

Despite these contributions, this review has several limitations. First, heterogeneity in both stigma measurement tools and intervention designs precluded quantitative meta-analysis of intervention effects on stigma reduction. Second, potential publication bias exists due to the inclusion of only English-language, peer-reviewed articles. Additionally, the geographic concentration of studies in South and Southeast Asia and Sub-Saharan Africa, while aligned with high-TB-burden regions, limits the generalizability of findings to high-income or low-incidence countries, which may experience different stigma dynamics and resource constraints. Finally, several of the included studies had methodological limitations, such as small sample sizes and reliance on non-randomized, qualitative, or cross-sectional designs, where self-reported stigma measures are vulnerable to social desirability bias and recall error.

The findings of this review have practical implications for TB control programs and policymakers. Interventions should be designed with explicit attention to the type(s) of stigma prevalent within the target population and should integrate multi-level strategies to ensure sustainability. The evidence supports investment in participatory and peer-led approaches, alongside broader health system reforms aimed at normalizing TB care and reducing institutional stigma. While educational campaigns remain essential, they must be culturally sensitive and embedded within existing community structures. Importantly, digital innovations, when designed with user experience in mind, can help mitigate anticipated stigma and enhance treatment adherence. These findings offer actionable recommendations for national TB programs and stakeholders seeking to achieve stigma-related targets in the WHO End TB Strategy.

## 5. Conclusions

In conclusion, the evidence presented in this review suggests substantial progress in both the development and implementation of TB stigma interventions over the past decade. The emergence of theoretically grounded, peer-led, and multicomponent approaches represents a critical advancement in addressing the complex sociocultural and structural determinants of TB-related stigma. Future research should aim to validate these findings through rigorous impact evaluations and explore scalability and sustainability of effective models across diverse settings.

## Figures and Tables

**Table 1:** Systematic review search strategy.

Database	Search Fields	Filters
PubMed	Title, Abstract, and Keywords	Language: English Date: 2015 to present
Scopus	Title, Abstract, and Keywords	Language: English Date: 2015 to present
Science Direct	Title, Abstract, and Keywords	Language: English Date: 2015 to present
ProQuest	Title, Abstract, and Keywords	Language: English Date: 2015 to present
Google Scholar	Title	Date: 2015 to present

**Table 2:** Inclusion and exclusion criteria of study selection based on PICOS framework.

PICOS Framework	Studies to Be Included	Studies to Be Excluded
Population	General public, adolescents, TB patients/survivors, caregivers, and healthcare providers	HIV patients and HIV/TB co-infected individuals
Intervention	TB-stigma-focused program development, TB treatment interventions demonstrating stigma reduction, and dedicated stigma reduction initiatives	TB treatment interventions without assessed stigma impacts
Comparator	Not applicable	Not applicable
Outcome	Stigma types addressed, target populations, and intervention components	TB treatment efficacy or knowledge outcomes without stigma metrics
Study design	Qualitative, quantitative, and mixed-methods studies	Reviews, editorials, commentaries, conference abstracts, and non-English publications

Abbreviations: HIV—human immunodeficiency virus; PICOS—Population, Intervention, Comparator, Outcome and Study Design; TB—tuberculosis.

**Table 3:** Risk of bias evaluation results.

Author, Year	Question 1	Question 2	Question 3	Question 4	Question 5	Question 6	Question 7	Assessment Results
Qualitative Studies	Clear RQ?	RQ Addressed?	Approach Appropriate?	Method Adequate?	Findings Adequate?	Results Substantiated?	Analysis Coherent?	Total “Yes” Answers
Patel, 2020 [18]	Yes	Yes	Yes	Can’t tell	Yes	Yes	Yes	6
Sabin, 2021 [19]	Yes	Yes	Yes	Yes	Yes	Yes	Yes	7
van der Westhuizen, 2024 [20]	Yes	Yes	Yes	Yes	Yes	Yes	Can’t tell	6
Mawey, 2023 [21]	Yes	Yes	Yes	Yes	Can’t tell	Can’t tell	Yes	5
Hayward, 2024 [22]	Yes	Yes	Yes	Yes	Yes	Yes	Yes	7
Mixed Methods Studies	Clear RQ?	RQ Addressed?	Rationale for MM?	Integration of Components?	Adequate Interpretation?	Divergences Addressed?	Quality Adherence?	Total “Yes” Answers
Wilson, 2016 [23]	Yes	Yes	Yes	Yes	Yes	Can’t tell	Yes	6
Foster, 2024 [24]	Yes	Yes	Yes	Yes	Yes	Yes	Yes	7
Fuady, 2025 [25]	Yes	Yes	Yes	Yes	Yes	Yes	Yes	7
Quantitative Descriptive Studies	Clear RQ?	RQ Addressed?	Sampling Relevant?	Sample Representative?	Measurement Appropriate?	Low Nonresponse Bias?	Analysis Appropriate?	Total “Yes” Answers
Taneja, 2017 [26]	Yes	Yes	Yes	Yes	Yes	Can’t tell	Yes	6
Kara, 2022 [27]	Yes	Yes	Yes	Yes	Yes	Can’t tell	Can’t tell	5
Moonsarn, 2023 [28]	Yes	Yes	Yes	Yes	Yes	Can’t tell	Yes	6
Macdonald, 2024 [29]	Yes	Yes	Yes	Yes	Yes	Can’t tell	Yes	6
Vibulchai, 2024 [30]	Yes	Yes	Yes	Yes	Yes	Can’t tell	Yes	6
Quantitative Non-Randomized Studies	Clear RQ?	RQ Addressed?	Participants Representative?	Measurement Appropriate?	Outcome Data Complete?	Confounders Accounted?	Intended Intervention Delivered?	Total “Yes” Answers
Idris, 2020 [31]	Yes	Yes	Yes	Yes	Yes	Can’t tell	Yes	6
Quantitative RCTs	Clear RQ?	RQ Addressed?	Randomization Appropriate?	Comparable Groups?	Outcome Data Complete?	Blind Assessment?	Participant Adherence?	Total “Yes” Answers
Faraade, 2023 [32]	Yes	Yes	Yes	Yes	Yes	Yes	Yes	7

Abbreviations: RCTs—randomized controlled studies; RQ—research question.

**Table 4:** Included study characteristics.

Author, Year	Country	Study Design	Methodology	Population	Outcome	Sample Size
Development of Stigma Interventions						
Sabin, 2021 [19]	India	Qualitative	In-depth interviews	Persons living with active TB	Intervention development	47 TB patients
Foster, 2024 [24]	South Africa	Mixed-methods	Mixed-methods	People affected with TB	Design and implementation of contextually relevant stigma reduction intervention	93 PWTB and 24 caregivers
Hayward, 2024 [22]	South Africa	Qualitative	Community-based participatory research approach	People diagnosed with TB, caregivers, and health workers	Co-development of person-centered stigma intervention	87 people diagnosed with TB, caregivers, and health workers
van der Westhuizen, 2024 [20]	South Africa	Qualitative	In-depth interviews	Health workers and PWTB	Recommendations for stigma reduction associated with TB IPC	18 HW and 15 PWTB
Fuady, 2025 [25]	Indonesia	Mixed-methods	Mixed-methods participatory action study	Relevant stakeholders with suitable and diverse TB expertise and/or experience and national participatory workshop with TB patients	Co-development of community-based, peer-led support intervention to reduce TB stigma	25 stakeholders and 20 TB patients
Tuberculosis Treatment-Related Interventions with Demonstrated Impact on Stigma Reduction						
Wilson, 2016 [23]	El-Salvador	Mixed-methods	Pilot intervention study with three-tier assessment	Health providers, TB patients, and family members	Improved TB and treatment knowledge among TB patients and their families	1916 TB patients and family members
Taneja, 2017 [26]	India	Quantitative	Quasi-experimental pilot study	Diagnosed MDR TB patients	Assessment of outcomes of Home-Based Care (intervention) versus No Home-Based Care (control) on treatment of MDR TB	50 intervention and 50 control
Patel, 2020 [18]	Uganda	Qualitative	Human-centered design	TB patients, family members, health workers, and community leaders	Identification of essential insights and potential areas for redesign of original 99DOTS platform	67 TB patients, family members, health workers, and community leaders
Stigma Reduction Interventions						
Idris, 2020 [31]	Malaysia	Quantitative	School-based, non-randomized controlled study	High school students	Improvement in TB knowledge, attitudes, practices, and stigma	236 students
Kara, 2022 [27]	Turkey	Qualitative	Cross-sectional	TB patients receiving directly observed therapy	Reduction in stigma	30 DOT and 52 VDOT TB patients
Faraade, 2023 [32]	Somalia	Quantitative	RCT	TB patients	Evaluation of effects of stigma intervention program	155 intervention and 150 control participants
Mawey, 2023 [21]	Indonesia	Qualitative	Case-report	MDR TB patient and public	Reduction in workplace stigma and depression	1 (case-report)
Moonsarn, 2023 [28]	Thailand	Quantitative	Quasi-experimental	Male high school students	Reduction in TB stigma and discrimination among high school students during COVID-19	216 students
Macdonald, 2024 [29]	Indonesia	Quantitative	Longitudinal intervention assessment	Trainers and TB patients and survivors	Reduction in TB self-stigma	22 TB patients
Vibulchai, 2024 [30]	Thailand	Quantitative	One-group within-subjects repeated-measure design	Patients with TB	Reduction in internalized stigma among patients with TB	26 TB patients

Abbreviations: DOT—directly observed therapy; HW—health workers; MDR—multidrug-resistant; PWTB—people with tuberculosis; RCT—randomized clinical trial; TB—tuberculosis; VDOT—video directly observed therapy.

**Table 5:** Types of stigma, intervention type, and components.

Author, Year	Stigma Type	Intervention Type and Components
Development of Stigma Interventions		
Sabin, 2021 [19]	Perceived, enacted, and internalized stigma	To address enacted stigma, interventions incorporated celebrity advocacy and school-based educational programs aimed at raising community awareness shifting public perception of tuberculosis. Support groups and counseling were recommended to reduce internalized stigma among people living with TB.
Foster, 2024 [24]	Anticipated, internalized, and enacted stigma	Community-engaged, multi-level, multicomponent interventions with context-specific adaptations and survivor-led messaging.
Hayward, 2024 [22]	Internalized, anticipated, enacted, and intersectional stigma divided into individual, interpersonal, institutional, community, and policy levels of experiences	Individual-level interventions include digital platforms enabling TB survivor peer support with structured messaging and integrated healthcare provider counseling. Interpersonal approaches involve peer-led experience sharing and family-centered counseling. At the institutional level, training healthcare workers with survivor insights enhances understanding of patient experience, while restructuring service delivery prioritizes patient-centered care. Community-level efforts focus on increasing TB awareness through targeted education and outreach initiatives. At the policy level, action is needed to evaluate and address how existing policies perpetuate stigma in order to promote inclusive healthcare practices.
van der Westhuizen, 2024 [20]	Anticipated, internal, enacted, and intersectional stigma using Link and Phelan’s theoretical model and the Health Stigma and Discrimination Framework	At the individual level, tailored patient education should comprehensively explain tuberculosis infection prevention and control protocols. At the interpersonal level, healthcare providers should communicate IPC guidelines to patients using respectful, non-stigmatizing language and approaches. At the institutional level, healthcare facilities should reframe IPC measures as community safety practices rather than fear-driven responses to infection. Within communities, efforts should focus on creating environments that support voluntary status disclosure by reducing anxiety about unintentional exposure through visible IPC practices. At the policy level, reforms should promote the adoption of universal airborne precautions in high-risk settings, extending beyond TB-specific protocols, to mitigate stigma while maintaining protection.
Fuady, 2025 [25]	Internalized, enacted, and anticipated stigma	The study developed individualized psychological evaluations and counseling sessions; monthly peer-facilitated group counseling interventions; peer-delivered one-on-one psychosocial support; and community-centered educational sessions on TB, referred to as “TB Talks.”
Tuberculosis Treatment-Related Interventions with Demonstrated Impact on Stigma Reduction		
Wilson, 2016 [23]	Anticipated and enacted stigma	The study developed a video-based TB education program comprising four key components: (1) general TB information, (2) TB screening and treatment protocols, (3) correction of common TB misconceptions, (4) authentic patient testimonials.
Taneja, 2017 [26]	Enacted stigma	The intervention comprised a home-based care system encompassing counseling, treatment adherence support, rehabilitative services, and nutritional assistance, delivered in conjunction with the standard pharmacological regimen.
Patel, 2020 [18]	Anticipated stigma	The study developed a human-centered redesign of the 99DOTS digital adherence technology, focusing on the pill packaging envelope displaying toll-free numbers for dose reporting and enhancing the overall patient experience with the system.
Stigma Reduction Interventions		
Idris, 2020 [31]	Perceived, enacted, and internalized stigma assessed using the KAPS questionnaire	The intervention comprised a school-based TB education program for high school students that included a lecture on TB symptoms and treatment, a quiz, small group discussions to address local myths and community attitudes and practices related to TB transmission and knowledge, and a poster exhibition promoting an anti-smoking campaign.
Kara, 2022 [27]	Internalized, enacted, and anticipated stigma	The study implemented video directly observed therapy for TB patients with the following components: video observation, asynchronous or synchronous communication, patient education and training.
Faraade, 2023 [32]	Community and patient stigma assessed with 11 questions	The intervention comprised video and lecture programs on stigma for TB patients.
Mawey, 2023 [21]	Enacted stigma	The study implemented workplace psychosocial intervention on TB spread, and patient education and counseling.
Moonsarn, 2023 [28]	Enacted, internalized, anticipated, perceived and secondary stigma	The educational intervention comprises four sequential modules: TB prevention knowledge, stigma awareness training, stigma reduction through cognitive restructuring and empowerment techniques, and sustained information support systems.
Macdonald, 2024 [29]	Self-stigma	The study deployed an anti-self-stigma toolkit based on a socio-ecological model, comprising eight modules: (1) self-stigma introduction, (2) shame coping strategies, (3) DR-TB-specific impacts, (4) infection control contexts, (5) health rights linkages, (6) treatment-related stigma, (7) post-TB future planning, (8) stigma impact evaluation.
Vibulchai, 2024 [30]	Internalized stigma	The peer-delivered intervention incorporated four components: TB-specific health education, psychoeducation, self-management training, and community-based home visits.

Abbreviations: DOT—directly observed therapy; DR-TB—drug-resistant tuberculosis; IPC—infection prevention and control; KAPS—knowledge, attitudes, and practice survey; TB—tuberculosis.

## Data Availability

The original contributions presented in this study are included in this article. Further inquiries can be directed to the corresponding author.

## Supplementary Materials

The following supporting information can be downloaded at https://www.mdpi.com/article/10.3390/healthcare13151846/s1. Table S1. PRISMA 2020 checklist for systematic reviews. Table S2. TB stigma assessment results.

## Abbreviations

The following abbreviations were used in this manuscript:

CI	Confidence Interval
DOT	Directly Observed Therapy
DR-TB	Drug-Resistant Tuberculosis
HW	Health Worker
IPC	Infection Prevention and Control
KAPS	Knowledge, Attitudes, and Practices Survey
MMAT	Mixed Methods Appraisal Tool
MDR	Multidrug-Resistant
PICOS	Population, Intervention, Comparator, Outcome, and Study Design
PRISMA	Preferred Reporting Items for Systematic Reviews and Meta-Analyses
PWTB	People with Tuberculosis
RCT	Randomized Controlled Trial
TB	Tuberculosis
VDOT	Video Directly Observed Therapy
WHO	World Health Organization
